# Dissecting the Single-Cell Diversity and Heterogeneity Underlying Cervical Precancerous Lesions and Cancer Tissues

**DOI:** 10.1007/s43032-024-01695-5

**Published:** 2024-10-01

**Authors:** Yanling Han, Lu Shi, Nan Jiang, Jiamin Huang, Xiuzhi Jia, Bo Zhu

**Affiliations:** 1 Department of Clinical Laboratory, Women’s Hospital, Zhejiang University School of Medicine, Hangzhou, Zhejiang 310006 China; 2 CRE Life Institute, Beijing, 100000 China; 3 Department of Immunology and Pathogen Biology, College of Medicine, Lishui University, Lishui, 323000 China; 4 Center of Disease Immunity and Intervention, College of Medicine, Lishui University, Lishui, 323000 China

**Keywords:** Cervical cancer, Tumor heterogeneity, Tumor microenvironment, Single-cell RNA-seq, ATAC-seq

## Abstract

The underlying cellular diversity and heterogeneity from cervix precancerous lesions to cervical squamous cell carcinoma (CSCC) is investigated. Four single-cell datasets including normal tissues, normal adjacent tissues, precancerous lesions, and cervical tumors were integrated to perform disease stage analysis. Single-cell compositional data analysis (scCODA) was utilized to reveal the compositional changes of each cell type. Differentially expressed genes (DEGs) among cell types were annotated using BioCarta. An assay for transposase-accessible chromatin sequencing (ATAC-seq) analysis was performed to correlate epigenetic alterations with gene expression profiles. Lastly, a logistic regression model was used to assess the similarity between the original and new cohort data (HRA001742). After global annotation, seven distinct cell types were categorized. Eight consensus-upregulated DEGs were identified in B cells among different disease statuses, which could be utilized to predict the overall survival of CSCC patients. Inferred copy number variation (CNV) analysis of epithelial cells guided disease progression classification. Trajectory and ATAC-seq integration analysis identified 95 key transcription factors (TF) and one immunohistochemistry (IHC) testified key-node TF (YY1) involved in epithelial cells from CSCC initiation to progression. The consistency of epithelial cell subpopulation markers was revealed with single-cell sequencing, bulk sequencing, and RT-qPCR detection. KRT8 and KRT15, markers of Epi6, showed progressively higher expression with disease progression as revealed by IHC detection. The logistic regression model testified the robustness of the resemblance of clusters among the various datasets utilized in this study. Valuable insights into CSCC cellular diversity and heterogeneity provide a foundation for future targeted therapy.

**Supplementary Information:**

The online version contains supplementary material available at 10.1007/s43032-024-01695-5.

## Introduction

Cervical cancer (CC) is the fourth most common gynecological malignancy, which is attributed to the progression of cervix precancerous lesions [1, 2]. Cervical squamous cell carcinoma (CSCC) accounts for approximately 80–85% of cases [3]. CSCC morbidity and mortality are global public health issues [4]. CSCC screening programs to identify the cervix precancerous lesions and human papillomavirus vaccination have reduced the incidence and mortality rates of CSCC [5–7]. Tumor heterogeneity presents a complex challenge in CSCC treatment, affecting therapeutic response, disease recurrence, and patient survival. However, the mechanism underlying tumor heterogeneity in CSCC and the time course change from cervix precancerous lesions to CSCC are not yet fully understood and warrant further research [8, 9].

Recent technological advancements, such as single-cell RNA-sequencing (scRNA-seq), have emerged as a powerful tool for providing granular transcriptome profiles of individual cells [10–12]. Several studies have employed single-cell analysis techniques to investigate the cellular heterogeneity and underlying mechanisms of CSCC [13, 14]. However, the limited sample size presents a considerable bottleneck, limiting the broader clinical use of scRNA-seq technology. Notably, the advantage of our study lies in the integration of multiple datasets, a feature unparalleled by current research efforts where such extensive sample data has not been previously undertaken.

An assay for transposase accessible chromatin sequencing (ATAC-seq) was primed to identify open chromatin regions, offering an epigenetic panorama by pinpointing active regulatory elements and binding sites for transcription factors to map nucleoside positions across the genome [15, 16]. Therefore, it is believed that integrating ATAC-seq data provides valuable insights into regulatory mechanisms, differentially regulated genes, and transcription factor interactions.

We aimed to comprehensively explore CSCC, focusing on its progression from precancerous cervical lesions to malignancy. We explored publicly available scRNA-seq data. We generated a dataset comprising 23 samples and 194,993 cells, which was the largest-scale single-cell study conducted to date on CSCC, to explore the relationship between its tumor microenvironment and the expression levels of differentially expressed genes in CSCC patients. We performed joint analyses alongside ATAC-seq data from The Cancer Genome Atlas Program (TCGA). In addition, we found that the patterns observed in our study are consistently present across different datasets, underscoring the reliability and reproducibility of our research. This study aimed to clarify the diversity and heterogeneity of CSCC development and to uncover its complex diversity through cell subpopulation characteristics and gene regulatory structures to identify potential biomarkers for novel diagnostic and treatment approaches.

## Materials and Methods

### Data Acquisition and Preparation

Data was obtained from four distinct 10× Genomics Single Cell 3′ v2 sequencing datasets sourced from GEO (GSE168652), the National Genomics Data Center (access number: PRJCA008573/HRA001742), and the EBI single cell database (E-MTAB-11948/E-MTAB-12305) (Table S1). The datasets incorporated a mixture of cervical tumors, normal adjacent tissues, normal tissues, and precancerous lesions. For dimensionality reduction, clustering, and scRNA-seq data analysis, we used Seurat (v4.1.1) software. We acquired ATAC-Seq information on Cervical Endocervical Adenocarcinoma and Squamous Cell Carcinoma (CESC) samples from The Cancer Genome Atlas (TCGA) repository, with ATAC-seq peaks obtained from the official TCGA publication website (https://gdc.cancer.gov/about-data/publications/ATACseq-AWG). Detailed steps and methodologies for data integration and annotation are described in the following sections. Genes expressed in at least three cells in each sample were included. Cells with < 200 unique molecular identifiers (UMIs) or > 20% of mitochondria-derived UMIs were deemed low-quality and were removed. Doublets were removed using the DoubletFinder package in R.

### Cell Composition Analysis

Cell-type compositional analysis statistics were calculated using a single-cell compositional data analysis (scCODA) package (v0.1.8)(https://github.com/theislab/scCODA) in Python (v3.9.2). Fresh samples were used as reference conditions for scCODA [17]. For comparison, a reference cluster with minimal variation among the samples was used. To maintain uniformity and replicability, scCODA was executed ten times using the Hamiltonian Monte Carlo sampling technique with default settings, and the outcomes were averaged. Cell types exhibiting average scores below (or above) zero were deemed to possess a notable decline (or increase) in abundance, as per the scCODA model (with a false discovery rate < 0.05).

### Differential Expression Gene (DEG) Analysis

We used the single-cell consensus optimization of DEG detection (scCODE) R package (v1.2.0.0) (https://github.com/XZouProjects/scCODE) to analyze DEGs in tumor/normal, tumor/precancer, and precancer/normal comparisons within each cell type. Significant genes were determined for all DEG analyses by the Bonferroni false discovery rate using the bilateral Wilcoxon rank-sum test. scCODE improves single-cell DEG analysis reliability by examining selected DEGs using various testing methods [18].

### Functional Enrichment Analysis

Functional enrichment analyses were performed using the clusterProfiler R package (version 4.1). Enriched Kyoto Encyclopedia of Genes and Genomes (KEGG) pathways were computed using DEGs in each cell type or cluster. The gene set variation analysis (GSVA) package was used to perform the GSVA. Preference gene sets for GSVA were selected from the Molecular Signatures Database (MSigDB), including gene sets from “c2.cp.biocarta.v2023.1.Hs.symbols.gmt”. Significantly enriched Biocarta pathways were selected by the LIMMA package.

### Pseudo-Time Trajectory Analysis

Pseudo-time trajectories were inferred using the Monocle2 package (version 2.30.0) to reveal the cell-state transitions. The “differentialGeneTest” function was performed using the default settings, and a q-value threshold < 1e^-10^ was set. The dimensionality of the dataset was reduced using the “reduceDimension” function with the DDRTree method. Specifically, the number of components was limited to a maximum of three, focusing on capturing the most significant variations in the gene expression data. Finally, the “orderCells” function was applied for cell ordering, allowing us to arrange cells according to their developmental trajectories or differentiation states.

### Copy Number Alteration (CNA) Inference

Python 3.8 infercnvpy was used to infer copy number variations (CNV) which discriminated malignant epithelial cells from nonmalignant epithelial cells [19]. We assumed all immune cell types to be non-cancer cells and used them as a reference to estimate the CNVs across all cell types. We identified malignant cells based on a threshold value of 0.025, and potentially malignant cells based on a threshold value of 0.01.

### Analysis of Key Genes and Transcription Factors Using ATAC-Seq

After identifying genes that consistently increased or decreased along the pseudo-time trajectories, we explored these genes further in ATAC-seq. ATAC-seq data for cervical cancer (CESC) samples were obtained from the TCGA. The type-specific CESC count matrices in the raw count files were processed to create BED-format files for each gene peak. The ChIPSeeker package was used to annotate the genomic location and proximity of each peak to known genes. The chromatin landscape of the selected genes and peak locations (bed) were further visualized using the WashU Epigenome Browser (http://epigenomegateway.wustl.edu/browser/). Human Transcription Factor genes, downloaded from AnimalTFDB4, were used for comparison with the results of ATAC-seq and pseudo-time variant genes to identify the key transcription factors.

### Cluster Similarity Analysis

To assess the resemblances between clusters in various datasets, we used elastic net regularization to train a logistic regression model. To avoid potential bias caused by varying sizes of training sets, we sampled each cluster to match the smallest cluster size in the training dataset prior to model training. Using these models, the anticipated logit for every cell in the test data for each cluster from the training data was computed, incorporating an offset value of 0. The predicted scores were averaged within each cluster and transformed into probabilities for visualization, revealing the resemblance between clusters in the test and training data [20, 21].

### Immunohistochemistry

The 2-um sections of normal cervical tissues, adjacent cervical tissues, precancerous lesions, and cervical tumors were derived from the biobank of Women’s Hospital, Zhejiang University School of Medicine. Antigen retrieval was performed by microwave-heating and 4% normal goat serum was utilized to block the nonspecific protein-binding. The sections were incubated with anti-human KRT8, KRT15, and YY1 rabbit polyclonal antibodies (1:1000; Proteintech Wuhan Sanying, Wuhan, China). Biotinylated mouse anti-goat Ig antibody, avidin: biotinylated enzyme complex, and 3,3′-DAP were used as detecting reagents (Zhongshan Goldenbridge Biotechnology). The slides were further counterstained with hematoxylin, and the images were scanned and projected with NDP.view2 Image viewing software (5X).

### Quantitative Real-Time RT-PCR (qRT-PCR) Analysis

Paraffin sections of CSCC patients (n = 5), precancerous lesions (n = 5), and healthy donors (n = 5) were obtained from the biobank of Women’s Hospital, Zhejiang University School of Medicine and were used for further qRT-PCR analysis. Total RNA isolation and complementary DNA (cDNA) amplification were performed as previously described [22]. Briefly, total RNA was extracted from the paraffin sections using an EZNA MicroElute Total RNA Kit (Omega Bio-Tek). 2µg total RNA was used for cDNA synthesis using oligo (dT) primers and AMV reverse transcriptase XL (Takara Bio, Shiga, Japan), and synthesized cDNA was used as a template for qRT-PCR analysis. The primer sequences were as follows: KRT10 (forward primer: 5′-CTTGGCAGAAACAGAAGGTCGCT-3′; reverse primer: 5′-GCAGGCTGCGGTAGGTTTGAA-3′), KRT1 (forward primer: 5′-CTTTTCTGCTGTTTCCCAATGAA-3′; reverse primer: 5′-GGAAAGAACAAAGCAGGGTCATAG-3′), KRT8 (forward primer: 5′-GGCTATGCAGGTGGTCTGAGC-3′; reverse primer: 5′-TTCCCATCACGTGTCTCGATCT-3′), KRT15 (forward primer: 5’-AGAAATCTGAATTCCTATTGCAGGAGA-3’; reverse primer: 5’-CCCTGAAAGCTTAGACCGAGGGACCCT-3’), YY1 (forward primer: 5′-AGAATAAGAAGTGGGAGCAGAAGC-3′; reverse primer: 5′-ACGAGGTGAGTTCTCTCCAATGAT-3′) and β-actin (forward primer: 5′-TCCTGTGGCATCCACGAAACT-3′; reverse primer: 5′-GAAGCATTTGCGGTGGACGAT-3′). Statistical analyses were carried out using R. *P* < 0.05 was considered statistically significant.

### Bulk RNA-seq Analysis

We applied the UCSC Xena Toil RNA-seq pipeline to quantify gene expression level, with hg38 as the reference genome and genecode V23 as the genomic annotation [23]. RSEM was used to quantitate gene expression [24].

### Statistical Analysis

R software (version 4.0.2) and Python (version 3.9) were used for statistical analysis and data visualization. Statistically significance was set to those with *p*-values or false discovery rate (FDR) values < 0.05, unless stated otherwise.

## Results

### Single-Cell Transcriptome Landscape in CSCC

**Fig. 1:**
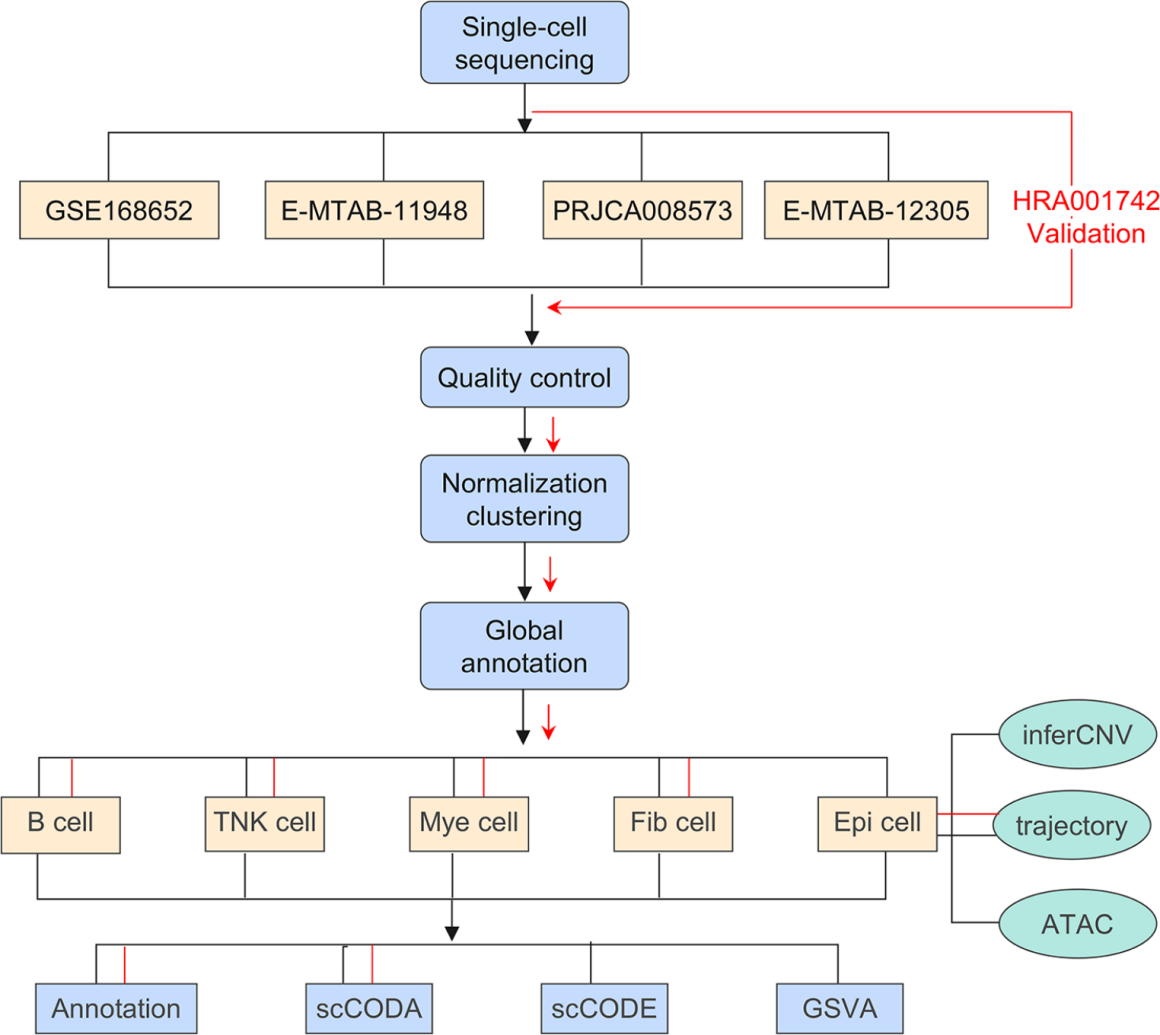
Study workflow. The collection of single-cell sequencing data from multiple datasets, including GSE168652, E-MTAB-11,948, PRJCA008573, and E-MTAB-12,305, with the last validation set. After data quality control, it is subjected to normalization and clustering for unique cell population control. Annotations are performed by global and subcluster levels within the datasets. Focused on subcluster levels, the analysis was performed using specialized computational tools (scCODA, scCODE, and GSVA). For epithelial cells (Epi), inferCNV is applied to infer copy number variations (CNV), and trajectory analysis in conjunction with ATAC-seq is used to elucidate the gene regulatory trajectories and chromatin accessibility patterns

**Fig. 2:**
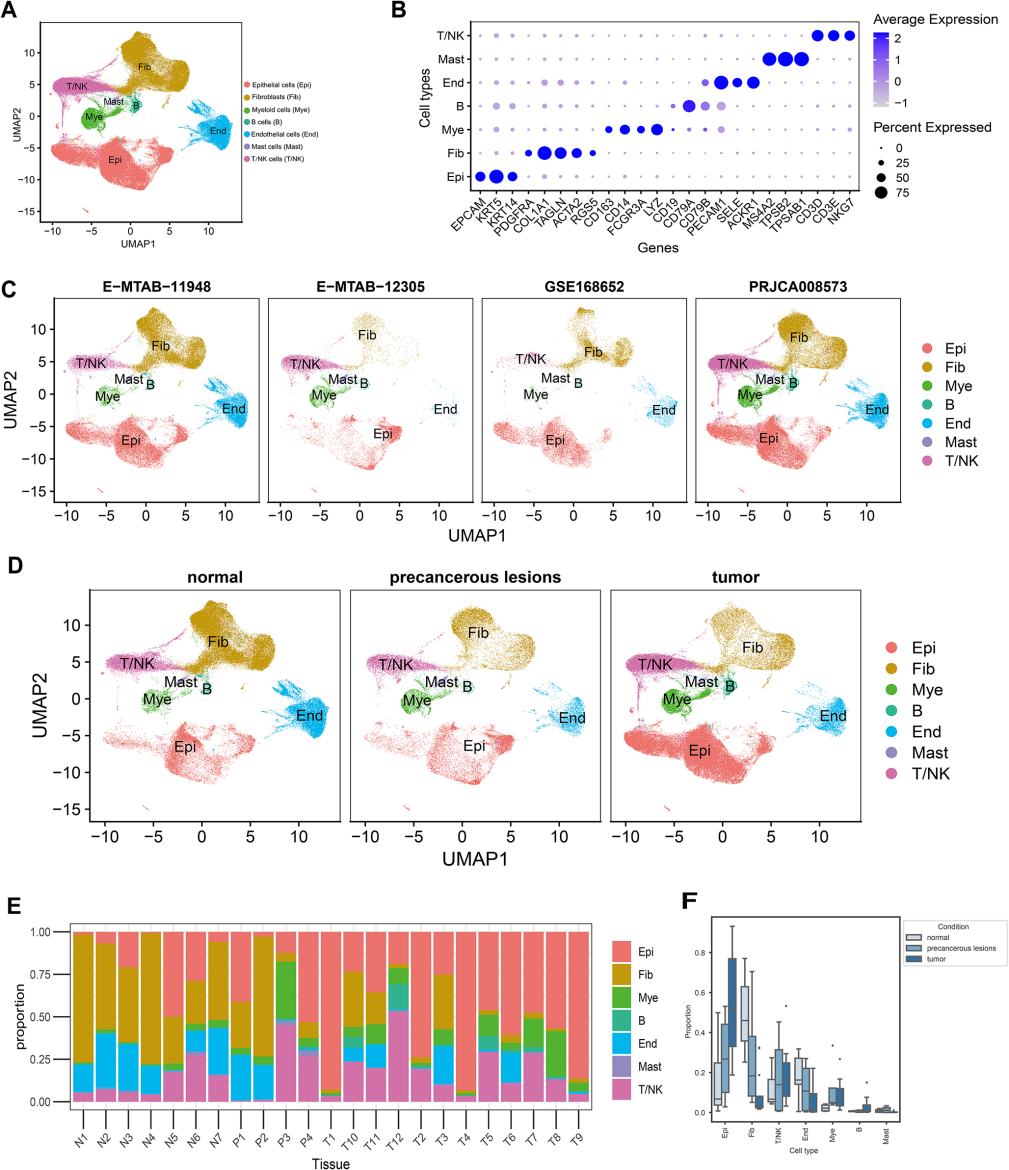
Single-cell landscape of cervical squamous cell carcinoma (CSCC). (**A**) All-identified cell clusters using the Uniform manifold approximation and projection (UMAP) method. Cell types are represented by different colors. (**B**) Heatmap showing expression levels of specific markers in each cell cluster. (**C**) Identified cell clusters are visualized using UMAP, grouped by dataset. (**D**) UMAP visualization of all identified cell clusters grouped by disease stage. (**E**) Cell type distribution in each sample. (**F**) Boxplots displaying the proportions of cell types at different disease stages, with significance determined by scCODA (*P* < 0.05)

The overall study flow scheme is depicted in Fig. 1, which shows the methodology used in this study. Single-cell sequencing data from multiple datasets, such as GSE168652 (one pair of cervical tumor tissues matched with their adjacent normal tissue data), E-MTAB-11,948 (three cervical tumors and three normal adjacent tissues), PRJCA008573 (eight cervical tumors, three normal cervical squamous tissues from the ectocervix, and two high squamous intraepithelial lesions), and E-MTAB-12,305 (two high squamous intraepithelial lesions) underwent quality control and normalization (Table S1). From the initial collection of 215,322 cells, 194,993 met the stringent quality control criteria. After global annotation, cells were categorized into seven distinct cell types (Fig. 2A). The optimal marker gene selection for cell type discrimination was demonstrated in Fig. 2B. The number and the proportion of distinct cell types were listed as follows: epithelial cells (72,226 cells, 37.04%, marked with *EPCAM*, *KRT5*, and *KRT14*), endothelial cells (23,877 cells, 12.25%, marked with *PECAM1*, *SELE*, and *ACKR1*), fibroblasts (54,342 cells, 27.87%, marked with *PDGFRA*, *COL1A1*, *TAGLN*, *ACTA2*, and *RGS5*), T/NK cells (28,895 cells, 14.82%, marked with *CD3D*, *CD3E*, and *NKG7*), B cells (3,190 cells, 1.64%, marked with *CD79A*, *CD79B*, and *CD19*), Myeloid cells (11,060 cells, 5.67%, marked with *CD163*, *CD14*, *FCGR3A*, and *LYZ*), and mast cells (1,403 cells, 0.72%, marked with *MS4A2*, *TPSB2*, and *TPSAB1*). Identified cell clusters grouped by different datasets were further visualized using UMAP (Fig. 2C), and identified cell clusters grouped by disease stage (normal tissue,75,805 cells, 38.88%; precancerous lesions, 30,120 cells, 15.45%; and tumor tissues, 89,068 cells, 45.68%) were also visualized with UMAP (Fig. 2D). Tumor samples exhibited a distinct cell lineage distribution from normal samples, with an increase in epithelial cells in the tumor samples, and a decrease in fibroblast cells (Fig. 2E). Furthermore, by considering the interdependence among subsets, we used the Bayesian scCODA model to examine alterations in the distribution of the seven cell types among the groups. The tumor samples exhibited a significant increase in the number of epithelial and B cells. Conversely, tumor samples showed a marked decline in fibroblast and endothelial cell counts. (Fig. 2F and Table S2).

### Single-Cell Expression Profile Characterization for Lymphocytes Across Disease Stage Groups

**Fig. 3:**
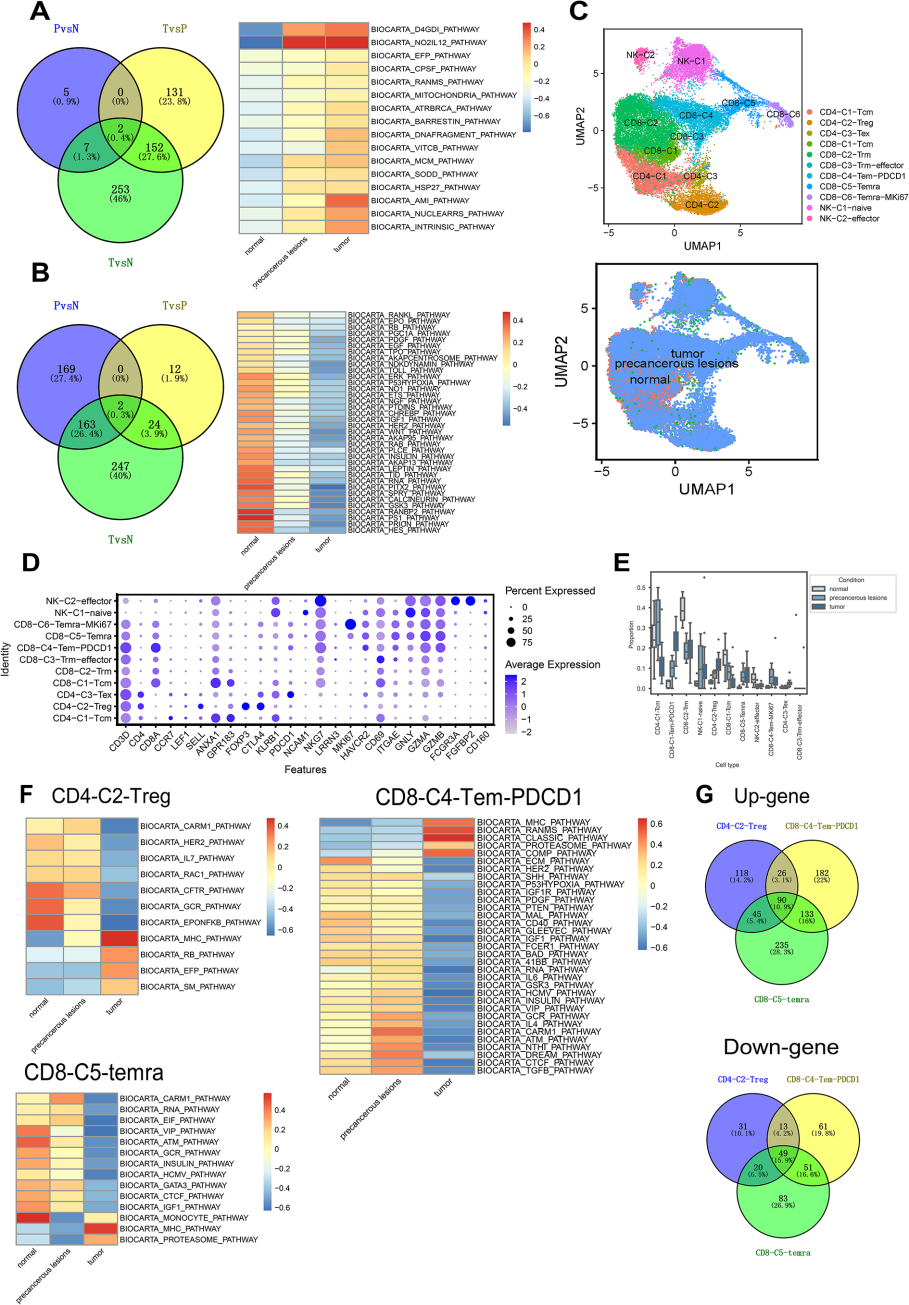
T and natural killer (NK) cell characteristics during cervical squamous cell carcinoma (CSCC) progression. (**A**) Venn diagram presenting the specific and common upregulated differentially expressed genes (DEGs) in B cells from normal, precancerous lesions, and tumor tissues (left panel). Heatmap indicating the expression level variations of upregulated genes in the Pathways for the three groups (right panel). (**B**) Venn diagram showing the specific and common downregulated DEGs in B cells from normal, precancerous lesions, and tumor tissues (left panel). Heatmap indicating the changes in the expression levels of downregulated genes in the Pathways pathway for the three groups (right panel). (**C**) UMAP visualization displaying all NK and T cells. Different colors represent cell types. (**D**) Heatmap showing the expression levels of specific markers in the T and NK cell subclusters. (**E**) Boxplots showing the proportions of T/NK cell subclusters at different disease stages, with significance determined using scCODA (*P* < 0.05). (**F**) Heatmap showing the expression level variations of CD4-C2-Treg, CD8-C4-Tem-PDCD1, and CD8-C5-temra in the Pathways for different disease stages. (**G**) Venn diagram showing the specific and common DEGs of CD4-C2-Tregs, CD8-C4-Tem-PDCD1, and CD8-C5-temra at different disease stages

To investigate the distribution and functional maps of B cells in CSCC, we isolated 3190 B cells. Notably, we found that B cells were significantly enriched in tumor tissue compared to normal tissue, and in the precancerous state. This suggests that subsequent B cell responses may be influenced by the tumor microenvironment (TME). Within the TME, mature B cells may process and present antigens, thus stimulating T cell activation. Additionally, they secrete cytokines such as IL-10 and TGF-β, which have anti-inflammatory effects in normal and tumor tissues, respectively. At the intersection of these three DEG sets, eight genes were identified as consensus upregulated DEGs: *HLA-DPA1*, *IGHA2*, *CD52*, *GNLY*, *LTB*, *TRBC2*, *GZMA*, and *HLA-DRB1*. Two genes, *LINC-PINT* and *SNHG5*, were downregulated. In the progression of the disease, we observed that certain apoptosis-related pathways were activated, such as role of mitochondria in apoptotic signaling, apoptotic DNA fragmentation and tissue homeostasis, SODD/TNFR1 Signaling, stress-induced HSP regulation and intrinsic prothrombin activation pathway, while other apoptosis-related pathways were inhibited, such as RB tumor suppressor/checkpoint signaling in response to DNA damage, p53-dependent apoptosis in ontogenically transformed cells, actions of Nitric Oxide and Erk1/Erk2 MAPK signaling pathway (Fig. 3A and B). This suggests a complex regulation of apoptosis during disease progression, where different pathways may be differentially regulated depending on the context and stage of the disease. It was worth noting that consensus upregulated B cell gene signature could be utilized to predict the survival of CSCC (Fig. S1).

T and NK cells also play crucial roles in the TME. Of the 25,244 T/NK cells, 55.04% (13,895 cells) were found in tumor tissues. NK cells are categorized into two subgroups: naive NK cells that highly express *NCAM1* and *GNLY*, and effector NK cells that highly express *GZMA*,* GZMB*,* NKG7*,* FCGR3A*, and *FGFBP2*. T cells, characterized by *CD3D*,* CD4*, and *CD8A*, are further classified into nine subclusters: three CD4 T subclusters, including CD4-C1 containing Central Memory CD4 T (Tcm) cells that highly express *CCR7*,* ANXA1*, and *GPR183*; CD4-C2 composed of Regulatory CD4 T (Treg) cells that highly express *LEF1*,* SELL*,* FOXP3*, and *CTLA4*; and CD4-C3 composed of exhausted CD4 T (Tex) cells that highly express *PDCD1* and *HAVCR2*. CD8 cells are segregated into six subclusters: CD8-C1 with Central Tcm cells highly express *CD8A*,* ANXA1*, and *GPR183;* CD8-C2 containing tissue-resident memory CD8 T (Trm) cells that highly express *CD8A*,* CD69*, and *ITGAE;* CD8-C3 incorporating Resident Memory CD8 T (Trm) cells with effector functions that highly express *CD8A*,* CD69*,* ITGAE*,* GZMA*, and *GZMB*; CD8-C4 composed of Effector Memory CD8 T (Tem) cells that highly express *PDCD1:CD8A*, and *PDCD1;* CD8-C5 consisting of terminally differentiated effector memory or effector CD8 T (Temra) cells that highly express *CD8A*,* KLRB1*, and *CD160;* and CD8-C6 including Effector Memory CD8 T (Temra) cells that highly express MKi67:*CD8A*, and *MIKI67* (Fig. 3C and D).

Within tumor tissues, there was a high enrichment of CD4 Treg, CD8-Tem-PDCD1, CD8-Temra, and CD4 + Tex cells (Fig. 3E and Table S3). We conducted DEG and gene set variation analysis (GSVA) for the CD4-C2-Treg, CD8-C4-Tem-PDCD1, and CD8-C5-Temra groups. The commonly affected signaling pathways across these groups included the BIOCARTA_MHC_PATHWAY (upregulated), BIOCARTA_GCR_PATHWAY (downregulated), and BIOCARTA_CARM1_PATHWAY (downregulated). The major histocompatibility complex (MHC) pathway manages antigenic peptide recognition and presentation, activates T cells, and initiates immune responses. The glucocorticoid receptor signaling (GCR) pathway is a nuclear receptor signaling pathway primarily involved in endocrine regulation, metabolism-related diseases, and endocrine tumor associations. Similarly, the BIOCARTA_CARM1_PATHWAY is a nuclear receptor signaling pathway mainly responsible for endocrine regulation, metabolic diseases, and endocrine tumor associations.

Both CD8-C5-Temra and CD8-C4-Tem-PDCD1 were enriched in inflammation, apoptosis, and cell proliferation-related pathways. CD4-C2-Treg was associated with T and B cell development, as well as cytoskeleton and cellular movement-related pathways. CD8-C5-Temra is linked with mRNA translation, Th2 cell cytokine gene expression, and macrophage formation. CD8-C4-Tem-PDCD1 is associated with the complement pathway, hypoxic stress, and cancer cells migration and invasion (Fig. 3F). Forty-three genes were identified in MHC, GCR, and CARM1 pathways. Furthermore, when merging the upregulated genes of CD4-C2-Treg, CD8-C5-Temra, and CD8-C4-Tem-PDCD1, nine intersected the GCR /MHC/CARM1 pathway. Among the genes downregulated in these groups, three intersected with the GCR /MHC/CARM1 pathway (Fig. 3G and Table S4).

### Single-Cell Expression Profile Characterization for Myeloid Cells Across Disease Stage Groups

**Fig. 4:**
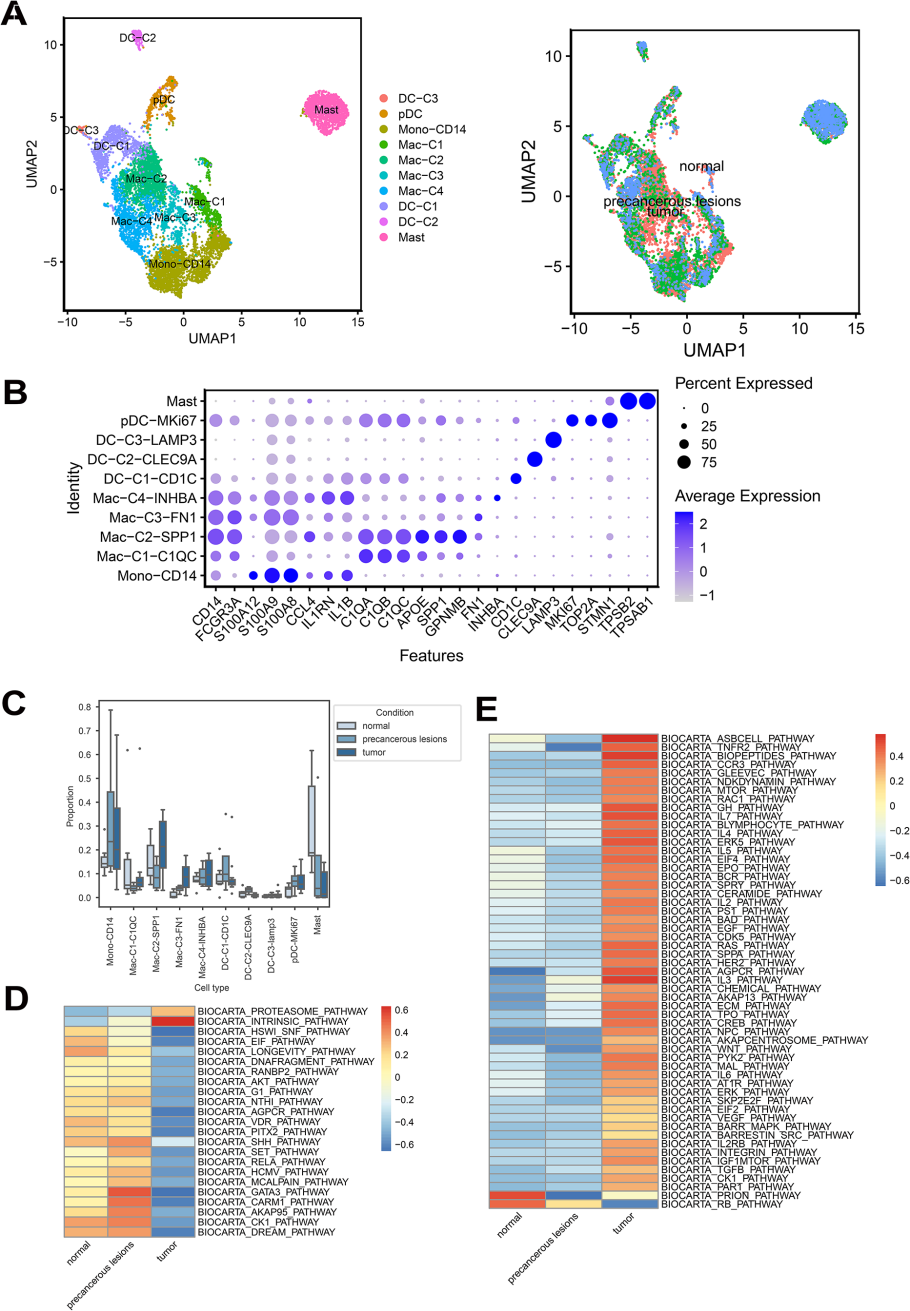
Myeloid cell characteristics during cervical squamous cell carcinoma (CSCC) progression. (**A**) UMAP visualization displaying all myeloid cells. Different colors represent cell types. (**B**) Heatmap illustrating the expression levels of particular indicators within subclusters of myeloid cells. (**C**) Boxplots illustrating the proportions of myeloid cell sub-clusters for different disease stages, with significance determined using scCODA (*P* < 0.05). (**D**) Heatmap displaying the changes in the expression levels of Mac-C3-FN1 in the Pathways across various disease stages. (**E**) Heatmap displaying the changes in the expression levels of mast cells in the Pathways across various disease stages

In CSCC, myeloid cells are a pivotal component of the immune cells that penetrate tumors and regulate tumor inflammation and angiogenesis. Of the 9,190 myeloid cells analyzed, 4,744 were normal (51.62%), 2,706 were precancerous (29.45%), and 1,740 were malignant, accounting for 18.93% of the total. Four main lineages, namely mast cells, plasmacytoid dendritic cells (pDCs), conventional dendritic cells (cDCs), and monocytes or macrophages, were distinguished within the TME using standard cellular markers. cDCs and groups encompassing monocytes or macrophages can be further subdivided. Specifically, in the cDC classification, we identified two traditional cDC subtypes (CD1C cDC1s and CLEC9A cDC2s) and one mature cDC subtype (LAMP3 cDC). Further clustering of the monocytes/macrophages yielded a monocyte subgroup (Mono-CD14) CD16 (FCGR3A), and four macrophage groups. Conventional macrophages were differentiated based on surface marker expression of *C1QC*, *INHBA*, *FN1*, and *SPP1* (Fig. 4A and B). To determine which of these subclusters was significant in the progression trajectory, we conducted a single-cell composition analysis of deformed cells (scCODA) across various disease stages. An increased proportion of FN1 ^+^ macrophage (as labeled in Fig. 4) was observed in different disease statuses from normal stage, precancerous stage, to tumor stage. While decreased proportion of Mast cells was observed in different disease statuses from tumor stage, precancerous stage, to normal stage (Fig. 4C and Table S5). Using GSVA, we enriched and compared divergent pathways. Mast cells were predominantly enriched in inflammatory response, angiogenesis, cellular activation, and proliferation-related pathways. In contrast, Mac-C3-FN1 was mainly enriched in chromosomal damage, cell cycle apoptosis, and nuclear receptor hormone-associated pathways (Fig. 4D and E).

### Single-Cell Expression Profile Characterization for Fibroblast Cells Across Disease Stage Groups

**Fig. 5:**
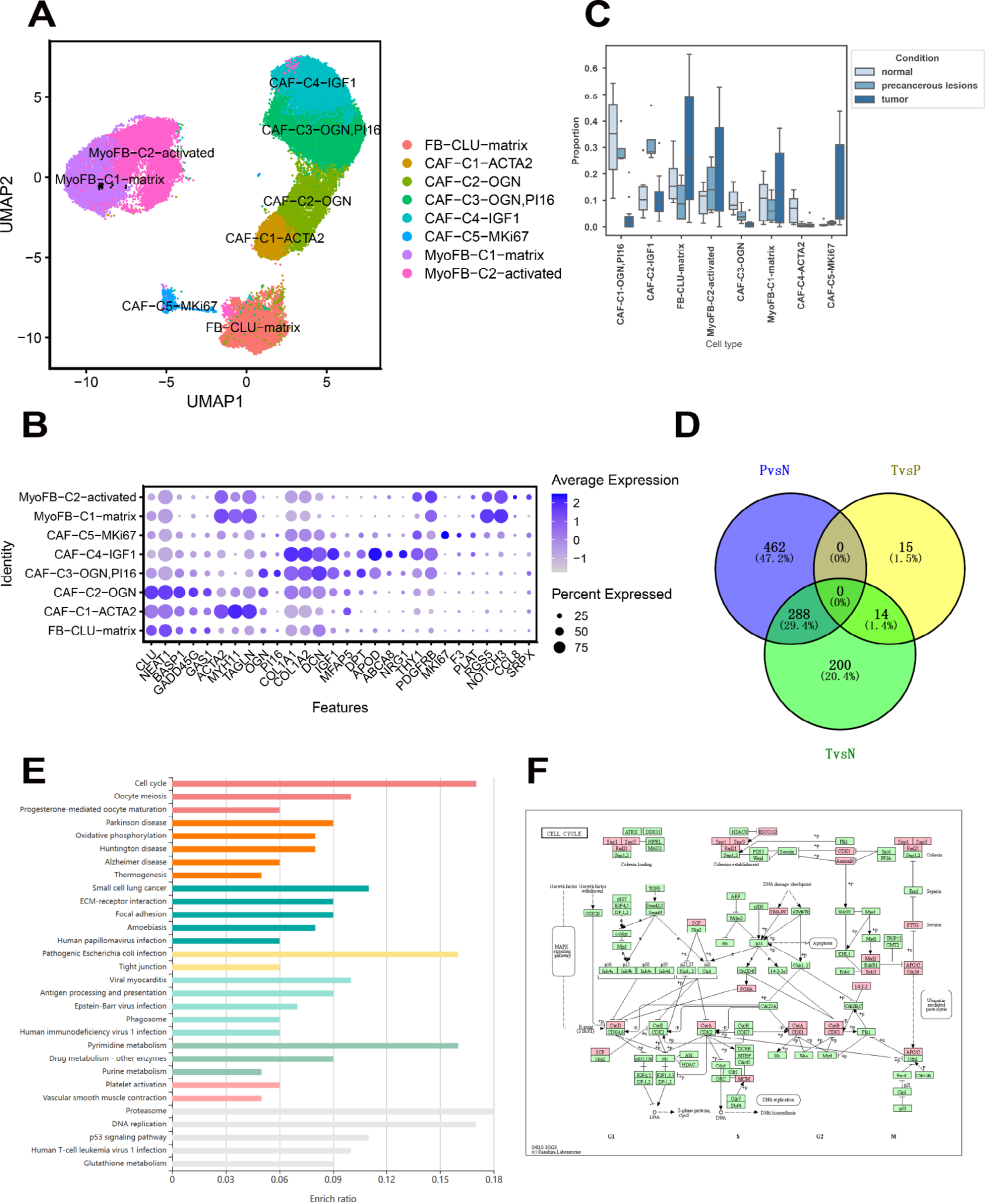
Fibroblast cell characteristics during cervical squamous cell carcinoma (CSCC) progression. (**A**) UMAP visualization of fibroblast cells with various colors indicating different cell types. (**B**) Heatmap presenting the cell type marker expression levels in the fibroblast subclusters. (**C**) Boxplots delineating the proportions of fibroblast subclusters among different disease stage groups, significance determined by scCODA (*P* < 0.05). (**D**) Venn diagram illustrating the specific and shared differentially expressed genes (DEGs) of the CAF-C5-MKi67 subcluster across the normal, precancerous lesion, and tumor stages. (**E**) Heatmap displaying the differential expression levels of CAF-C5-MKi67 in the Kyoto Encyclopedia of Genes and Genomes (KEGG) database across the three disease stages. (**F**) Cell cycle pathway depicted in the KEGG pathway maps

Fibroblasts can be broadly categorized into three main types: Normal fibroblasts (FB-CLU-matrix), characterized by the marker *CLU*,* NEAT1*, and *BASP1*, which maintains tissue structural integrity and participates in the wound repair process in normal tissues; and myofibroblasts that are further divided into two subtypes: MyoFB-C1-matrix which is identified by *ATAC2*,* MYH11*,* TAGLN*,* PDGFRB*,* RGS5*, and *NOTCH3*, and MyoFB-C2-activated by *ATAC2*,* MYH11*,* TAGLN*,* THY1*,* PDGFRB*,* RGS5*, and *NOTCH3*. These factors influence tumor cell migration and invasion. Cancer-associated fibroblasts (CAF) are divided into five subclusters: ACTA2 with markers *ATAC2*,* MYH11*, and *TAGLN;* OGN with markers *CLU*,* NEAT1*,* BASP1*,* GADD45G*,* GAS1*, and *OGN*; OGN/P16 with markers *CLU*,* NEAT1*,* BASP1*,* GADD45G*,* GAS1*,* OGN*, and *P16;* IGF1 with markers *COL1A1*,* COL1A2*,* DCN*,* IGF1*,* APOD*,* ABCA8*, and *NRG1;* and MKI67 with *MKI67*,* F3* markers (Fig. 5A and B). The MKI67 subcluster proportions increased substantially with a concurrent decline in the OGN and OGN/P16 subclusters (Fig. 5C and Table S6). Subsequent DEG and KEGG pathway analyses indicated that alterations in the MKI67 sub-cluster were associated with cell cycle regulation, cell adhesion, immune responses, hormonal regulation, and HPV infection (Fig. 5D and E, and Fig. 5F).

### Epithelial Cell Characteristics During CSCC Progression

**Fig. 6:**
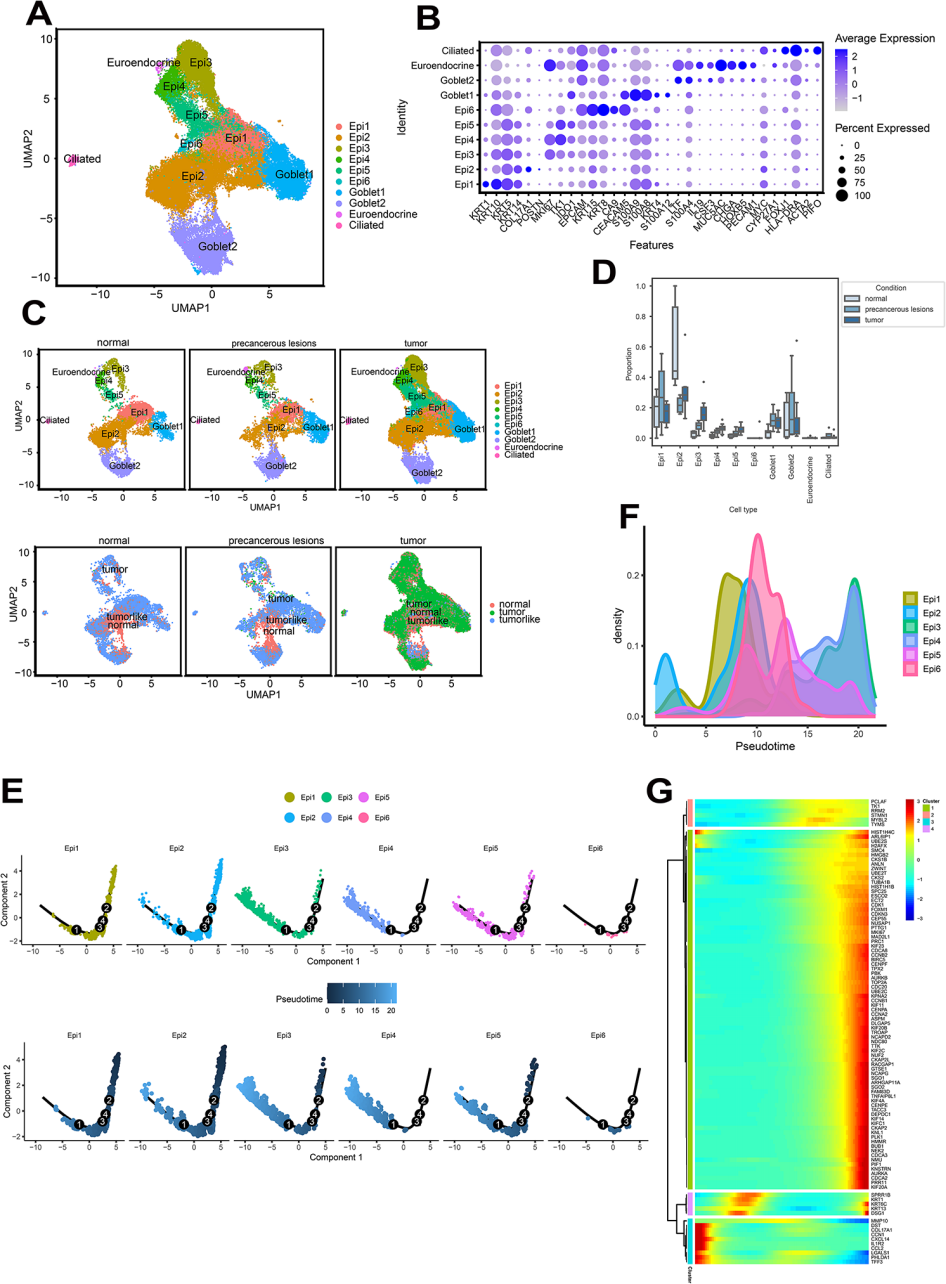
Epithelial cell characteristics during cervical squamous cell carcinoma (CSCC) progression. (**A**) Epithelial cell visualization using UMAP, with colors indicating distinct cell types. (**B**) Heatmap depicting specific marker expression levels in the epithelial subclusters. (**C**) UMAP visualization of epithelial sub-clusters categorized by group (upper panel). Inferred copy number variation (CNV) levels of epithelial cells, using B, mast, Mye, natural killer T(NKT), fibroblast, and end cells as references (lower panel). (**D**) Boxplots showing the proportions of epithelial sub-clusters across different disease stage groups, with significance determined using scCODA (*P* < 0.05). (**E**) Trajectory analysis of epithelial cells. Epithelial cell differentiation trajectories inferred using monocle v.2, with cells color-coded by identified clusters to illustrate the progression through various differentiation states over pseudotime. (**F**) Differential gene expression heatmap. This heatmap displays the top 100 differentially expressed genes (DEGs) along the inferred pseudotime trajectory, highlighting genes with significant expression changes that may contribute to cell state transitions. (**G**) Gene regulation in identified clusters. Expression regulation patterns within clusters: Clusters c1 and c2 are characterized by upregulated gene expression, indicating active cellular processes or differentiation states, whereas cluster c4 shows a downregulation pattern, which may reflect a distinct cellular function or quiescent state

With epithelial cell clustering, we identified cell subtypes, including ciliated epithelial, neuroendocrine, and goblet cells. Epithelial cells(Epi) were subsequently subdivided into six distinct sub-clusters (Fig. 6A and B). In contrast to normal tissues, we observed diminished Epi1 and Epi2 distribution in tumor tissues, whereas those of Epi3, Epi4, Epi5, and Epi6 increased (Fig. 6C and D, Table S7, and Fig.S2, S3). These data suggest that during the differentiation of normal cells into cancerous cells, there was a marked shift in the epithelial cell expression patterns. We used Monocle 2 for the pseudotemporal analysis to delve deeper into these alterations. The findings positioned Epi1 and Epi2 at the starting point of the trajectory, with Epi3, Epi4, Epi5, and Epi6 located towards the end (Fig. 6E and F). This may reflect the progression of development from normal to malignant cells. Further differential gene expression testing using Monocle 2 revealed changes in the expression of 5,648 genes throughout this process. These genes clustered into five groups. Notably, clusters c1 and c2 included genes that consistently showed increased expression over the pseudotemporal trajectory, whereas cluster c4 included genes that consistently exhibited decreased expression. These continually shifting genes may play significant roles in the carcinogenesis process (Fig. 6G, Fig. S4, and Table S8).

### Sc-RNA Sequencing Trajectory and ATAC-seq Peak Analysis Reveals Dynamic Chromatin Accessibility

**Fig. 7:**
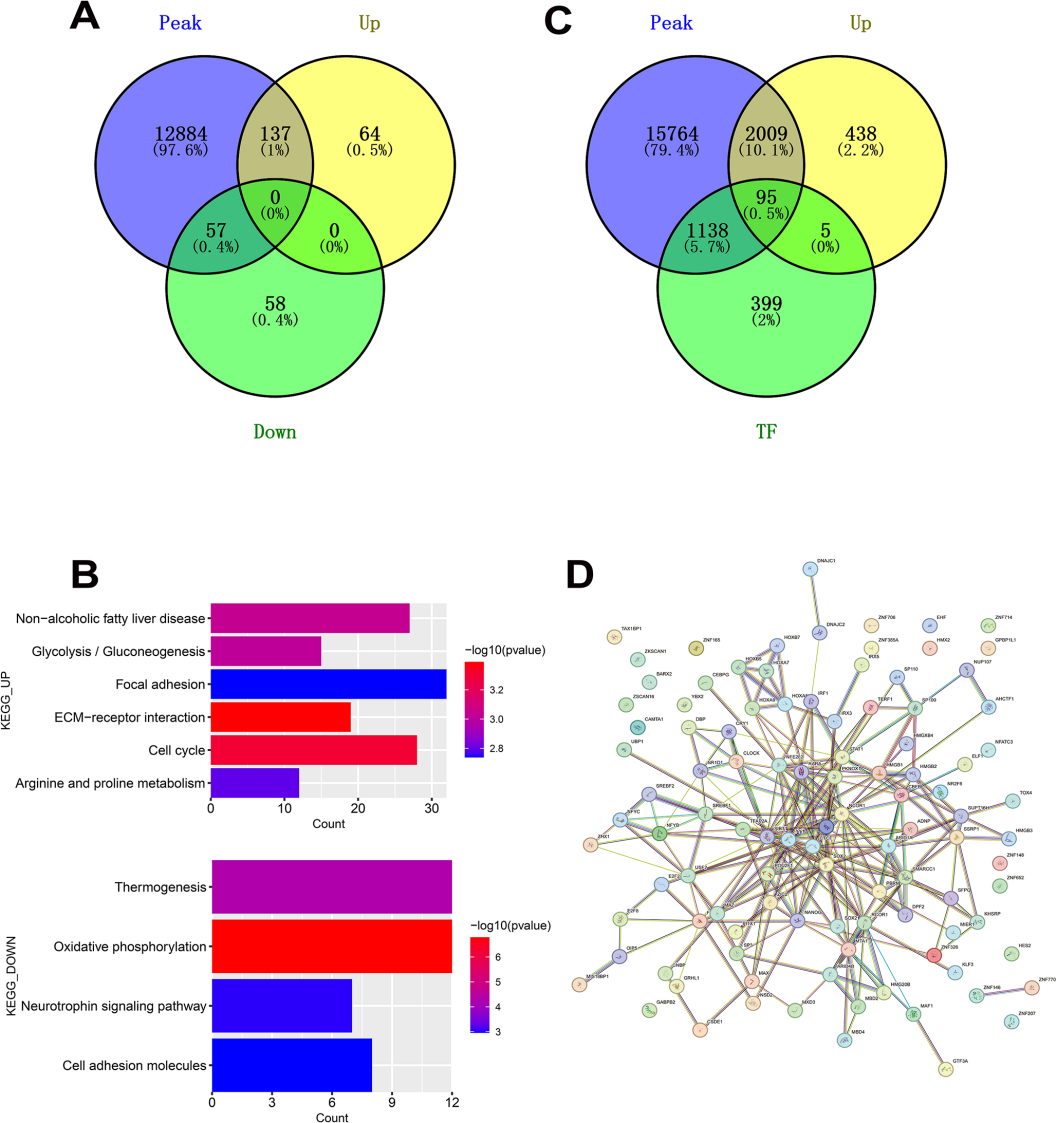
ATAC sequencing characteristics. (**A**) Venn diagram illustrating the distinct and overlapping genes among the three categories: DE_pseudotime upregulated, DE_pseudotime downregulated, and ATAC-seq peaks. (**B**) KEGG pathways enriched among shared genes between DE_pseudotime upregulated and ATAC-seq peaks (upper panel) and DE_pseudotime downregulated and ATAC-seq peaks (lower panel). (**C**) Venn diagram showing specific and overlapping genes in three categories: DE_pseudotime upregulated, transcription factors (TF), and ATAC-seq peaks. (**D**) Protein-protein interaction (PPI) network associated with 95 intersecting genes

ATAC-seq analysis provides insights into open chromatin regions, highlighting the active genomic regions in cells. To elucidate the relationship between DEGs over pseudotime (DE_pseudotime) and the chromatin landscape, we integrated the data from DE_pseudotime and ATAC-seq peaks. The distances from the peaks to the nearest transcription start site (TSS) were found to range between − 2,000 and 1, 137 DE_pseudotime upregulated genes showed changes in the ATAC peaks, suggesting a direct correlation between gene upregulation and chromatin accessibility. Conversely, of the DE_pseudotime-downregulated genes, 57 exhibited alterations in the ATAC peaks. (Fig. 7A). To understand the biological importance of these alterations, KEGG pathway analysis was conducted on the shared genes. The upregulated genes were primarily involved in ECM-receptor interaction, cell cycle, glycolysis/gluconeogenesis, arginine and proline metabolism, and focal adhesion. In contrast, the downregulated genes were mainly centered around pathways such as thermogenesis, oxidative phosphorylation, neurotrophin signaling, and cell adhesion molecules (Fig. 7C). We extended our analysis to explore the potential transcriptional regulators of these pseudotime DEGs. By comparing the ATAC-seq peaks to known transcription factors (TFs) and upregulated genes, we identified 95 intersecting genes (Fig. 7B and Table S9). They focus on chromatin regulation/acetylation, Notch signaling pathway, and MAPK Signaling Pathway. A protein-protein interaction (PPI) of 95 genes resulted in an interaction network that regulated the upregulated gene pathways (Fig. 7D and Table S10).

### Comprehensive Validation of Cell Clusters and Target Genes

**Fig. 8:**
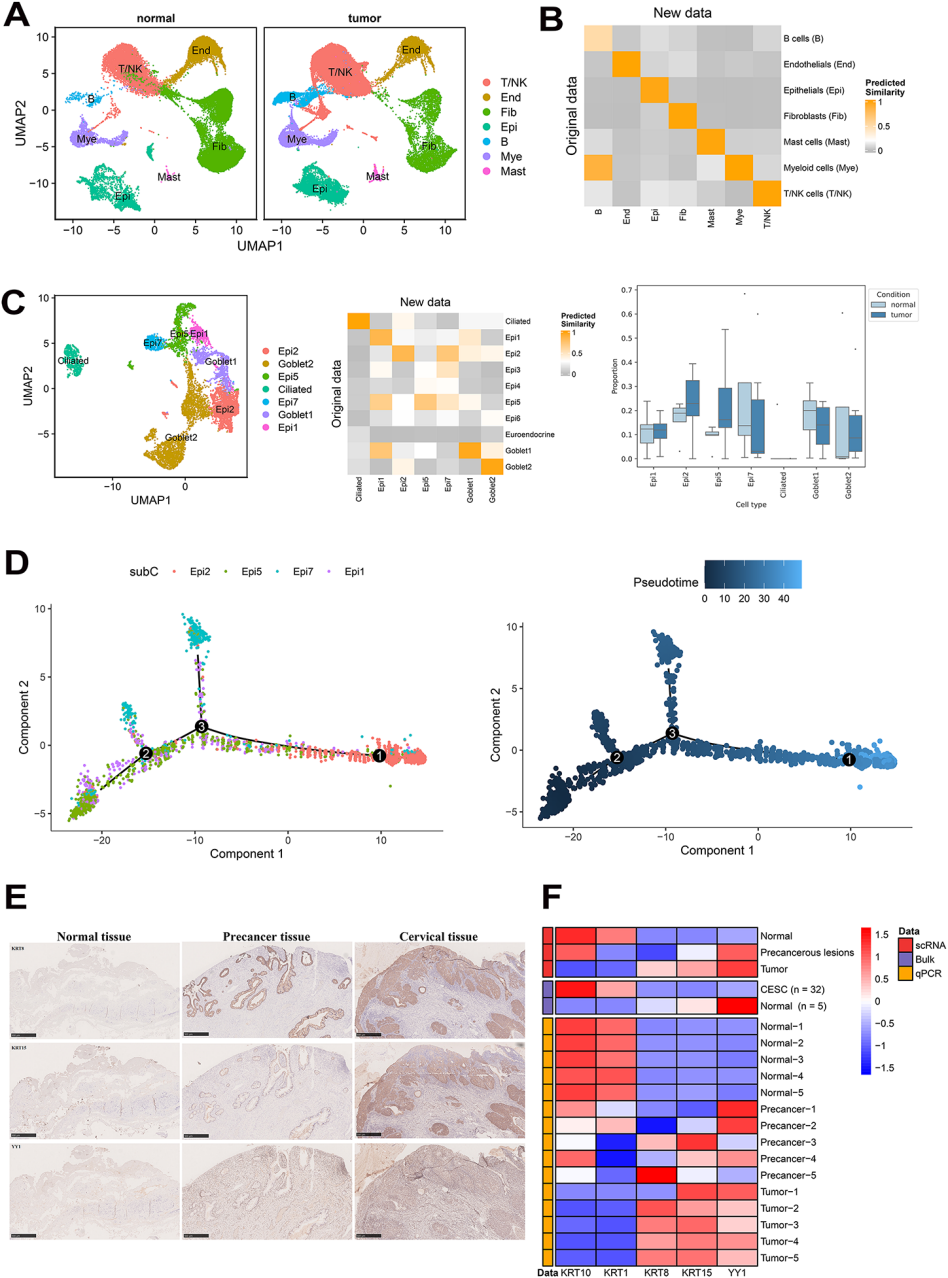
Validation of our findings by other datasets and methods. (**A**) Visualization of all cell clusters was performed using the UMAP for new datasets (HRA001742). Cell types are represented by different colors. (**B**) Similarities analysis of all identified cell clusters from original and new data. (**C**) UMAP representation of epithelial cells in the new data set (left panel). Similarities between epithelial cells from the original data and new data (middle panel). Boxplots showing the epithelial sub-cluster proportions of disease stage groups in the new data; significance was determined by scCODA (*P* < 0.05)(right panel). (**D**) The developmental trajectory of epithelial cells in the new data. (**E**) Representative example of a CSCC tumor stained by IHC with Immunohistochemical Analysis of KRT8, KRT15, as the cell marker Epi6, and Transcription Factor YY1. All images shown were captured at the same magnification (400x); the scale bars represent 500 microns. (**F**) Representative examples of the gene expression, such as cell markers KRT1 and KRT10 in the Epi1 subset, cell markers KRT8 and KRT15 in the Epi6 subset, and transcription factor YY1 of Epi1. Heatmap of logFC showing the expression as determined by our scRNA-seq (top), bulk RNA-seq (middle), and RT-qPCR experiments (bottom) across normal, precancerous, and CESC samples. All displayed genes showed significant differences (FDR < 0.05)

The tumor and normal adjacent tissue cell types from the new data (HRA001742) were also annotated accordingly (Fig. 8A). We used a logistic regression model with elastic net regularization to assess the similarity between the clusters in the original datasets (GSE168652, E-MTAB-11948, PRJCA008573, and E-MTAB-12305) and the new datasets (HRA001742). It was found that the degree of similarity of global cell types across datasets is very high (Fig. 8B). The number of cells in the new dataset is significantly lower than in the original dataset, making it impossible to annotate all cell types present in the original dataset. However, a large proportion of cells are still identifiable when delving into sub-clusters of T/NK cells, myeloid cells, and fibroblast cells. For example, we identified regulatory CD4 T (Treg) cells and memory CD8 T (Temra) cells that highly express MKi67 in the new dataset, both of which showed a clear increase in tumor tissues. The new data also revealed a higher enrichment of mast cells in tumor tissues, and nearly all fibroblast subtypes were identified, except for OGN ^+^ CAF (Fig. S5). Attention should be paid to the epi clusters. Epi1 and Epi2 represent the normal epithelium, whereas Epi7 is a unique cancer epithelium found only in the new data. Epi6 is a cancer epithelium exclusive of the original data, demonstrating strong cancer epithelium specificity (Fig. 8C). This indirectly emphasized the pronounced specificity of epithelial cells, which can be influenced by patient-specific characteristics. Our pseudotemporal analysis also confirmed this, indicating that Epi1 and Epi2 were in the early stages, whereas Epi5 and Epi6 were relatively later (Fig. 8D). This further emphasizes the advantages of multi-data integration, which enables smaller subgroup identification that may be overlooked in single or smaller datasets, leading to detailed discoveries.

On the other hand, a biological experiment was also performed to verify the finding. IHC assay confirmed at the protein level that the relative expression of KRT8 and KRT15 (Epi6 cell markers) increased progressively with the disease stage. Such staining also indicated the increased proportion of Epi6 cells during the development of cervical cancer, which could be the future target. The expression of the transcription factor YY1 was elevated in both the precancerous and tumor groups compared to normal tissues (Fig. 8E). Additionally, the consistency of epithelial cell subpopulation markers was revealed with single-cell sequencing, bulk sequencing, and RT-qPCR detection (Fig. 8F and Table S11). All of these results demonstrated the robustness of our results.

## Discussion

The CSCC treatment complexity is mainly attributed to tumor diversity and heterogeneity, which significantly affects treatment response, disease recurrence, and patient survival [25]. In this study, we extensively examined different cellular subcategories, such as lymphocytes, myeloid, fibroblast, and epithelial cells, to uncover their dynamic changes and interactions during disease progression. Moreover, we identified potentially crucial molecules and pathways that play significant roles in CSCC development and its ability to elude the immune system. The discoveries offer a valuable understanding of the biological processes that underlie CSCC.

Lymphocytes play a crucial role in the TME. In our study, as the disease progressed, the *IGHG4*, *HLA-DPA1*, *IGHA2*, *CD52*, *GNLY*, *LTB*, *TRBC2*, *GZMA*, and *HLA-DRB1* expression related to B cells were upregulated, whereas *LINC-PINT* and *SNHG5* were downregulated. In accordance with our finding that consensus upregulated B cell genes could be utilized to predict the overall survival of CSCC, *HLA-DPA1*, *IGHA2*, *CD52*, *HLA-DRB1*, and *GZMA* are testified as prognostic markers for gynecological tumors, providing a theoretical basis for their use as targets in clinical immunotherapy [26–28]. LINC-PINT, a long intergenic non-coding RNA triggered by p53, inhibits tumor cell proliferation in glioblastoma, hepatocellular carcinoma, and laryngeal squamous cell carcinoma (LSCC), making it a potential therapeutic target for tumor treatment [29–31].

In recent years, there have been an increasing number of studies on T cells in tumors, and the comprehensive blueprint of T cells has become more detailed. In our study, we found that CD4 Treg, CD8-Tem-PDCD-1, and CD8-Temra levels gradually increased during tumor progression. The nature of the immune response is determined, and the balance between T effector cells and T regulatory cells is crucial for the outcome [32]. Activated effector T cells infiltrate the tumor bed and kill cancer cells. Effector memory T cells and tissue-resident memory T cells are the main pathways for exhausted T cells, with effector memory T cells expressing high levels of the inhibitory receptor PDCD1 gradually transitioning to an exhausted state called “transitional cells”, reducing the anti-tumor function of other effector T cells [33, 34]. Additionally, these cells may have tumor-specific TCRs, suggesting an overall immune reaction. Temra is Tem-derived, and TCR similarity analysis has indicated that these two groups share TCR clonotypes. Zhang et al. showed that Temra cells in the CD4^+^ and CD8^+^ compartments of the pan-cancer single-cell atlas of tumor-infiltrating T cells show significant expansion in all tissues but have a lower proliferation rate. Individuals with cancer have a greater ratio of CD8Temra cells in their blood than those who are in good health. These expanded cells may possess tumor-specific TCRs, indicating a systemic immune response. CD8Temra cells proliferate within the tumor and migrate outside, demonstrating their high migratory potential between peripheral blood and cancer-adjacent/tumor tissues [35]. This highlights their roles in systemic immune responses. Our analysis identified common signaling pathways that affected all three groups: BIOCARTA_MHC_PATHWAY (upregulated), BIOCARTA_GCR_PATHWAY (downregulated), and BIOCARTA_CARM1_PATHWAY (downregulated). MHC pathway upregulation suggests T cell-mediated immune responses, emphasizing the importance of antigen presentation in cancer. Moreover, GCR and CARM1 pathway downregulation may be associated with tumor growth and survival, as these pathways are involved in endocrine regulation and metabolic diseases.

Mast cell activation in pathways involving inflammatory responses, angiogenesis, and cell activation and proliferation may have a substantial impact on tumor progression and immune regulation [36]. As most tumors contain inflammatory cell infiltration, including a large number of mast cells, there may be a pathogenic relationship between MC, chronic inflammation, and cancer [37]. Compared to other tumor myeloid studies, we identified FN1 as a unique CSCC subcluster (Fig. S6). The presence of FN at tumor locations is essential for acquiring the biological abilities required for different cancer traits, including sustaining proliferative signaling, stimulating angiogenesis, aiding invasion and metastasis, controlling growth suppression, and adjusting antitumor immunity [38, 39]. The increased Mac-C3-FN1 expression was linked to pathways associated with chromosomal damage, cell cycle regulation, and programmed cell death, indicating that these cells may play a role in suppressing tumor growth or facilitating tumor cell apoptosis.

MKI67, a cell proliferation marker, is upregulated in cancer. Crucial regulators and essential gene identification associated with cell proliferation and cell cycle can provide valuable guidance for the advancement of successful therapeutic approaches targeting CSCC [40]. Moreover, angiogenesis-linked pathway activation is associated with the pathological progression of CSCC, as the invasive stages exhibit elevated gene expression levels associated with these pathways. Targeting these pathways with drugs may prove effective.

In our study, we clarified the transformation trajectory of epithelial cells and combined them with ATAC data, which play a key role in metabolic regulation, energy production, biosynthetic needs, and proliferation of tumor cells, which are closely related to tumor development and progression [41, 42]. We also studied the potential transcriptional regulators of these DE_pseudotime genes and identified 95 transcription factors that may interact with these genes. The PPIs observed between these transcription factors and the upregulated genes provide insight into gene regulation within cells. It is worth noting that as the hub node transcription factor, YY1 regulates the expression of viral oncogenes E6 and E7 during HPV infection, and is also involved in the tumorigenesis process [43–45]. However, half of the downregulated genes showed changes in ATAC peaks, which may be due to the heterogeneity of tumor samples or changes in experimental conditions.

KRT8 and KRT15 increase progressively with the development of cervical cancer. KRT8^high^ alveolar intermediate cells are important intermediate state cells in the development of lung adenocarcinoma (LUAD) and are closely associated with KRAS mutations and tumor formation [46, 47]. KRT15 plays a significant role in the progression from high-grade intraepithelial neoplasia to esophageal squamous cell carcinoma, which is associated with tumor initiation and early development. In esophageal carcinoma, the high expression of KRT15 is closely related to tumor invasiveness, metastasis, and poor prognosis [48, 49]. All of these demonstrated the important neoplastic transformation role of KRT8 and KRT15 in tumors. While the importance of KRT8 and KRT15 is rarely reported in cervical cancer, these findings offer opportunities to identify new therapeutic targets and develop targeted treatment strategies.

Our study also emphasizes the importance of multi-dataset analysis. By comparing different datasets, we validated our findings and revealed inter-patient heterogeneity. This approach not only strengthens the reliability of our conclusions but may also help identify cell subgroups that might be overlooked in individual datasets. However, this study has some limitations. Firstly, our single-cell study included 13 tumor patients, and the limited number of samples may not fully represent the heterogeneity present in larger populations, potentially affecting the generalizability of the results. Second, although immunohistochemistry and RT-qPCR provided important validation, their scope is limited compared to other potential methods, such as functional assays or protein-level quantification across larger cohorts.

Overall, our study offers a comprehensive view of the heterogeneity and immune surroundings of cervical precancerous lesions and cancer tissues. By conducting a detailed analysis of various cell subpopulations, we identified cellular heterogeneity and potential therapeutic targets in CSCC. The unique characteristics of these cells, especially Epi6 cells, offer novel insights into tumor progression.

## Electronic Supplementary Material

Below is the link to the electronic supplementary material.

Supplementary Material 1

Supplementary Material 2

## Data Availability

Data for this study can be found in the Gene Expression Omnibus (GEO, https://www.ncbi.nlm.nih.gov/geo/), the National Genomics Data Center (https://ngdc.cncb.ac.cn/), and the European Bioinformatics Institute (EBI, https://www.ebi.ac.uk/biostudies/arrayexpress/studies).

## Abbreviations

CC: Cervical cancer
CSCC: Cervical squamous cell carcinoma
CESC: Cervical Endocervical Adenocarcinoma and Squamous Cell Carcinoma
scRNA-seq: Single-cell RNA-sequencing
DEGs: Differentially expressed genes
scCODA: Single-cell compositional data analysis differentially expressed genes
scCODE: Single-cell consensus optimization of DEG detection
GSEA: Gene Set Enrichment Analysis
GSVA: Gene Set Variation Analysis
ATAC-seq: Transposase accessible chromatin sequencing
TCGA: The Cancer Genome Atlas Program
UMIs: Unique molecular identifiers
CNV: Copy number variation
CNA: Copy number alteration
GO: Enriched gene ontology
KEGG: Kyoto Encyclopedia of Genes and Genomes
MSigDB: Molecular Signatures Database
UMAP: Uniform manifold approximation and projection
TME: Tumor microenvironment
Tcm: Central Memory T
Treg: Regulatory T
Tex: Exhausted T
Trm: Tissue-resident memory T
Temra: Terminally differentiated effector memory or effector T
MHC: Major histocompatibility complex
GCR: Glucocorticoid receptor signaling
TSS: transcription start site
DE_pseudotime: DEGs over pseudotime
TFs: Transcription factors
PPI: Protein-protein interaction

## Code Availability

Code could be provided with a request.
