# Supplementary material for: Dissecting the Single-Cell Diversity and Heterogeneity Underlying Cervical Precancerous Lesions and Cancer Tissues
Source: Reprod Sci. 2024 Oct 1;32(5):1502–19. doi: 10.1007/s43032-024-01695-5 (PMC12041141; doi:10.1007/s43032-024-01695-5)
Supplement: Supplementary file 1 — Supplementary Material 1 [file 43032_2024_1695_MOESM1_ESM.docx]

**Title**:**Dissecting the single-cell diversity and heterogeneity underlying cervical precancerous lesions and cancer tissues**

**Journal**: Journal of Reproductive Sciences

**Authors**:Yanling Han,^1^† Lu Shi,^2^† Nan Jiang,^1^ Jiamin Huang,^1^ Xiuzhi Jia,^3,4^* BO Zhu^1^*

^1^Department of Clinical Laboratory, Women’s Hospital, Zhejiang University School of Medicine, Hangzhou, Zhejiang, 310006, China

^2^CRE Life Institute, Beijing, 100000, China

^3^Department of Immunology and Pathogen Biology, College of Medicine, Lishui University, Lishui, 323000, China

^4^Center of Disease Immunity and Intervention, College of Medicine, Lishui University, China, Lishui, 323000, China

***Corresponding authors**

Xiuzhi Jia

E-mail address : [jiaxiuzhi@lsu.edu.cn](mailto:jiaxiuzhi@lsu.edu.cn)

BO Zhu

E-mail address : 5202054@zju.edu.cn

The list for Supplementary File 1 is as follows: Fig. S1-Fig. S6.


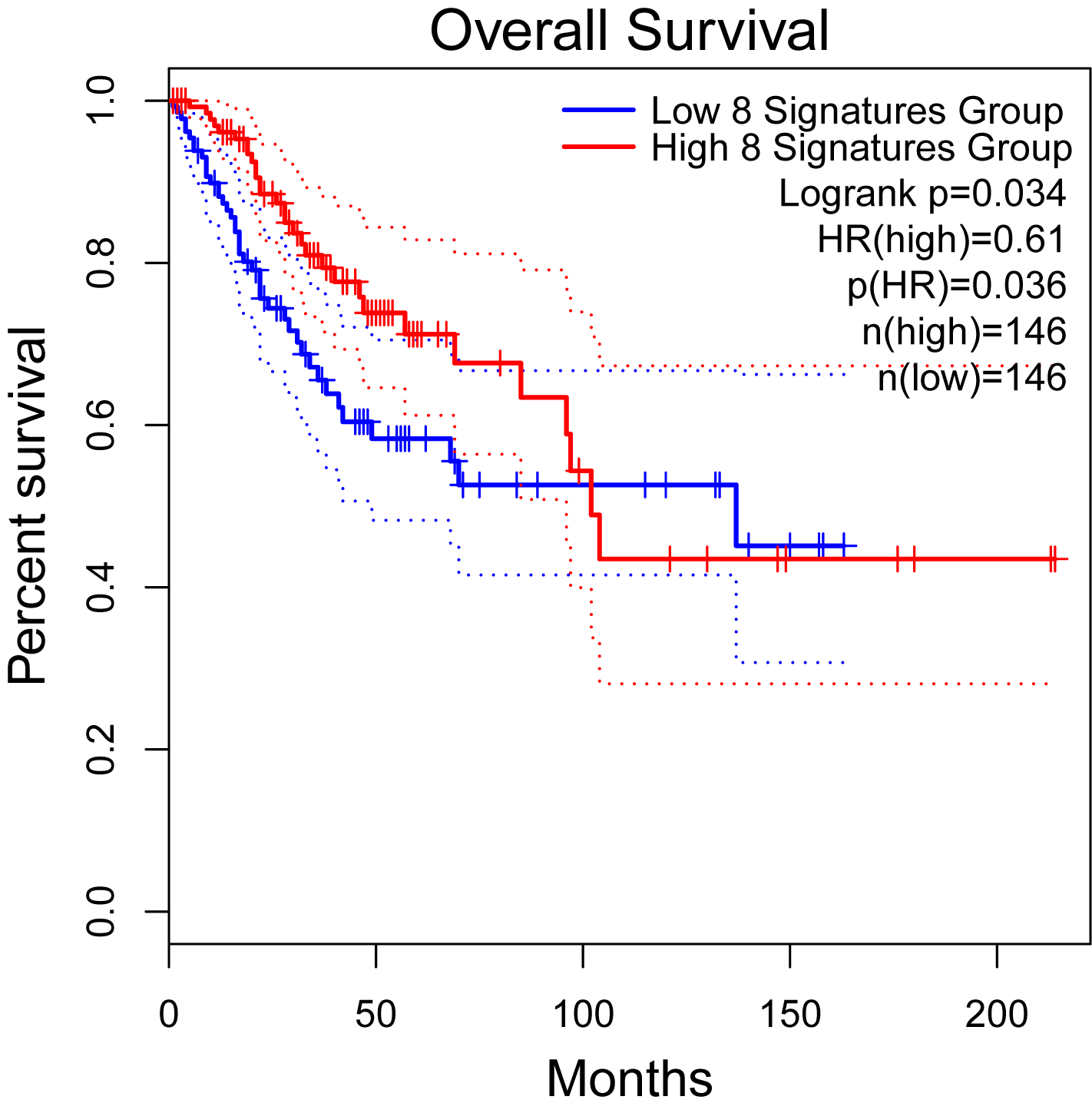


**Fig. S1** Overall survival based on 8 signature groups. The Kaplan-Meier curves illustrate the overall survival of patients stratified into two groups based on the expression of 8 specific signatures, derived from the GEPIA database(http://http://gepia.cancer-pku.cn/).


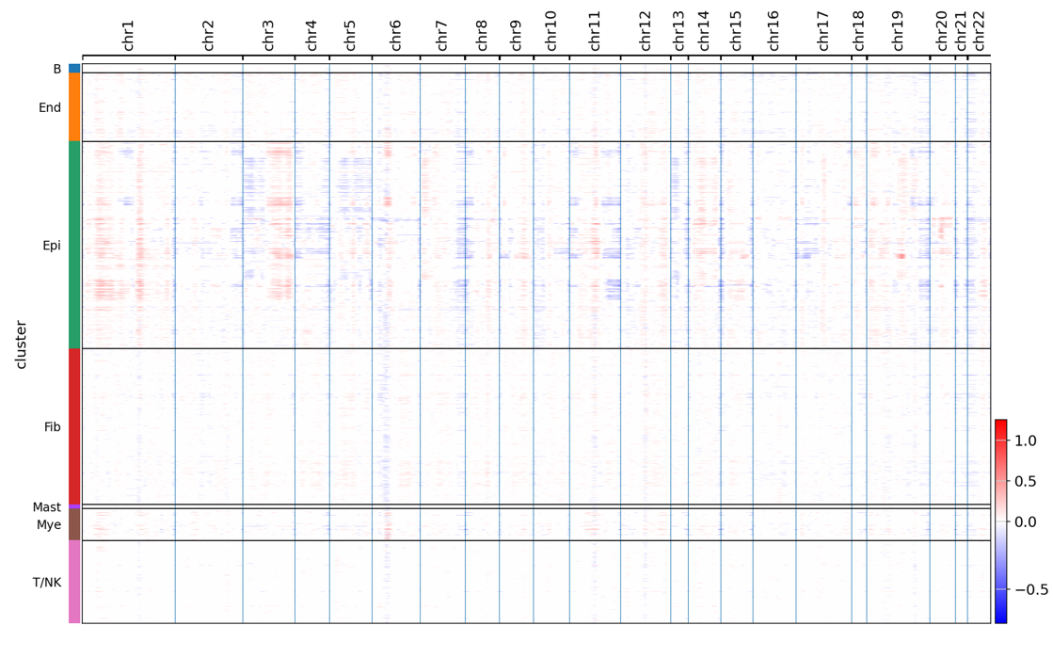


**Fig. S2** Heat maps of the CNV distribution of different chromatin of different cells. Blue indicates low modified expression, corresponding to genomic loss; red indicates high modified gene expression, corresponding to genomic gain. reference_cat=[ "B", "Mast", "Mye", "T/NK", "Fib", "End" ]


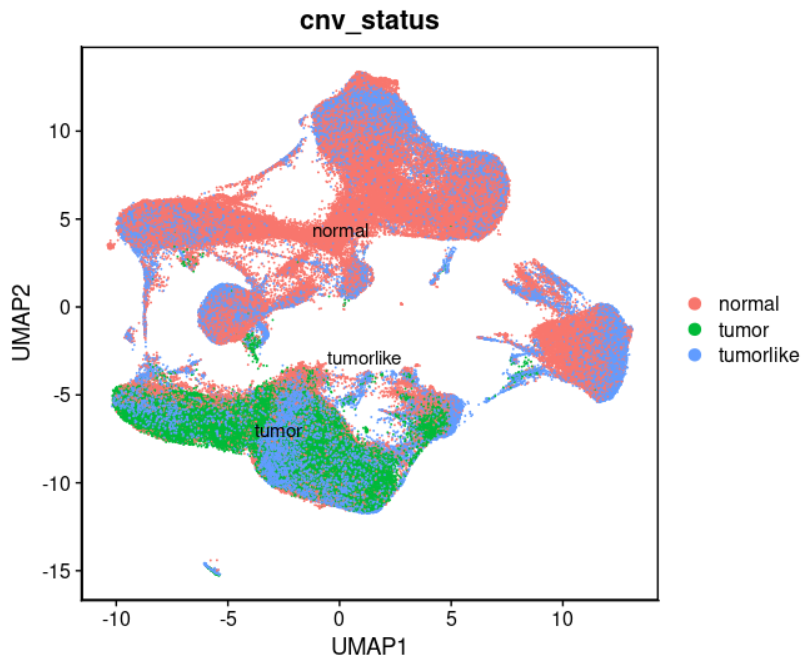


**Fig. S3** Copy number variation (CNV) status using the UMAP method. Different colors represent the CNV status: red, blue, and green for normal, tumor, and tumor-like cells, respectively


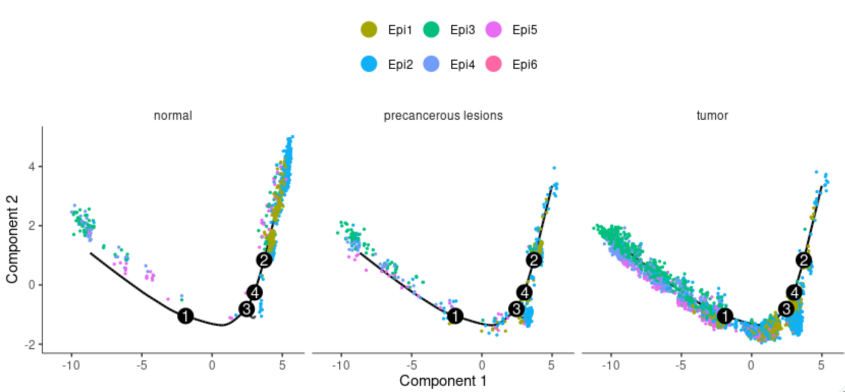


**Fig. S4** Distribution of the six subclusters of epithelial cells, grouped by disease stage


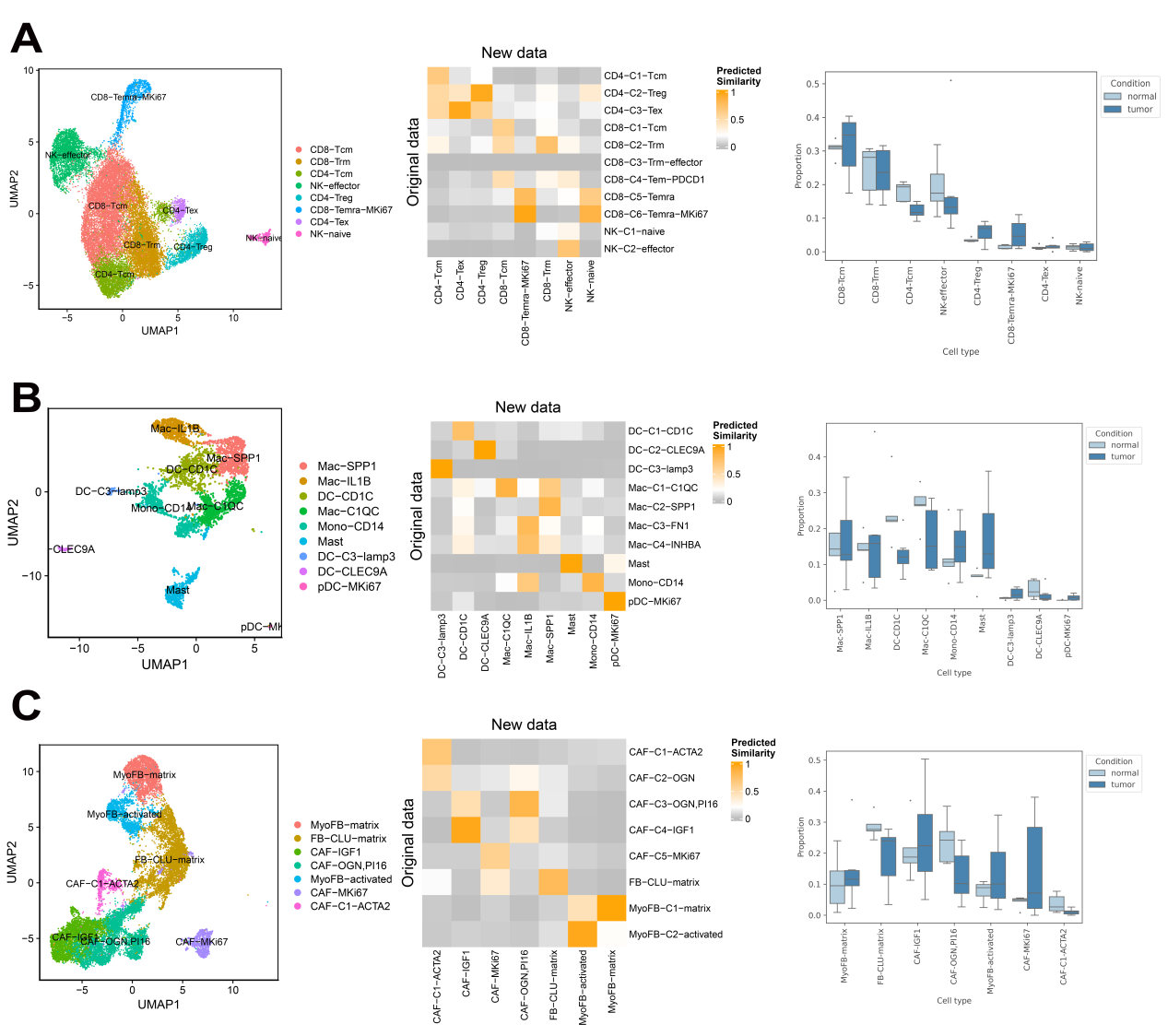


**Fig.S5**

A UMAP visualization of all natural killer (NK) and T cells in the new data (left panel). Similarities between NK and T cells from the original and new data (middle panel). Boxplots showing the T/NK subcluster proportions of disease stage groups in the new data, significance determined by scCODA (P < 0.05) (right panel).

B UMAP visualization of myeloid cells in the new data (left panel). Similarities between myeloid cells from the original data and new data (middle panel). Boxplots showing the myeloid subcluster proportions of disease stage groups in the new data, significance determined by scCODA (P < 0.05)(right panel).

C UMAP display of fibroblast cells in the new data (left panel). Comparison of fibroblast cells between the original and new data (middle panel). Boxplots showing the fibroblast subcluster proportions of disease stage groups in the new data, significance determined by scCODA (P < 0.05)(right panel).


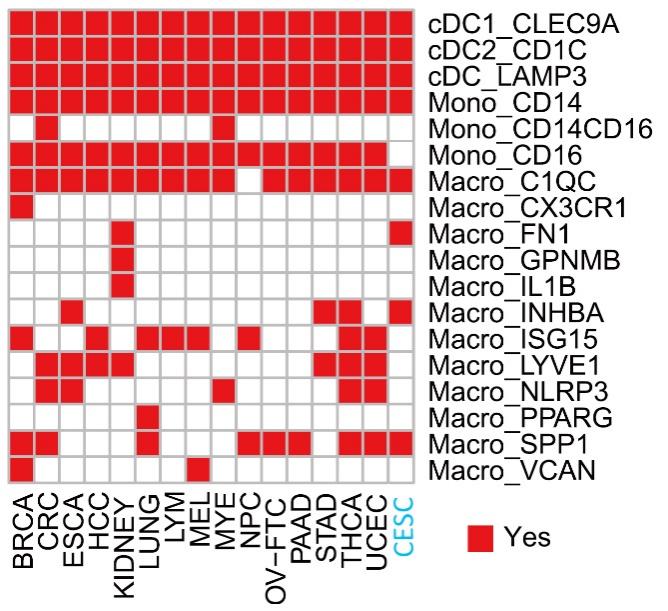


**Fig. S6** Myeloid cell subpopulation comparisons in cervical squamous cell carcinoma with the A pan-cancer single-cell transcriptional atlas (Zhang et al)
